# Are pangolins the intermediate host of the 2019 novel coronavirus (SARS-CoV-2)?

**DOI:** 10.1371/journal.ppat.1008421

**Published:** 2020-05-14

**Authors:** Ping Liu, Jing-Zhe Jiang, Xiu-Feng Wan, Yan Hua, Linmiao Li, Jiabin Zhou, Xiaohu Wang, Fanghui Hou, Jing Chen, Jiejian Zou, Jinping Chen

**Affiliations:** 1 Guangdong Key Laboratory of Animal Conservation and Resource Utilization, Guangdong Public Laboratory of Wild Animal Conservation and Utilization, Guangdong Institute of Applied Biological Resources, Guangdong Academy of Science, Guangzhou, Guangdong Province, China; 2 Key Laboratory of South China Sea Fishery Resources Exploitation & Utilization, Ministry of Agriculture, South China Sea Fisheries Research Institute, Chinese Academy of Fishery Sciences, Guangzhou, Guangdong Province, China; 3 Department of Molecular Microbiology and Immunology, School of Medicine, University of Missouri, Columbia, Missouri, United States of America; 4 Department of Electrical Engineering & Computer Science, College of Engineering, University of Missouri, Columbia, Missouri, United States of America; 5 MU Center for Research on Influenza Systems Biology (CRISB), University of Missouri, Columbia, Missouri, United States of America; 6 Bond Life Sciences Center, University of Missouri, Columbia, Missouri, United States of America; 7 MU Institute for Data Science and Informatics, University of Missouri, Columbia, Missouri, United States of America; 8 Guangdong Provincial Key Laboratory of Silviculture, Protection and Utilization, Guangdong Academy of Forestry, Guangzhou, Guangdong Province, China; 9 Institute of Animal Health, Guangdong Academy of Agricultural Sciences, Guangzhou, Guangdong Province, China; 10 Guangdong Provincial Wildlife Rescue Center, Guangzhou, Guangdong Province, China; University of Iowa, UNITED STATES

## Abstract

The outbreak of a novel corona Virus Disease 2019 (COVID-19) in the city of Wuhan, China has resulted in more than 1.7 million laboratory confirmed cases all over the world. Recent studies showed that SARS-CoV-2 was likely originated from bats, but its intermediate hosts are still largely unknown. In this study, we assembled the complete genome of a coronavirus identified in 3 sick Malayan pangolins. The molecular and phylogenetic analyses showed that this pangolin coronavirus (pangolin-CoV-2020) is genetically related to the SARS-CoV-2 as well as a group of bat coronaviruses but do not support the SARS-CoV-2 emerged directly from the pangolin-CoV-2020. Our study suggests that pangolins are natural hosts of *Betacoronaviruses*. Large surveillance of coronaviruses in pangolins could improve our understanding of the spectrum of coronaviruses in pangolins. In addition to conservation of wildlife, minimizing the exposures of humans to wildlife will be important to reduce the spillover risks of coronaviruses from wild animals to humans.

## Author summary

Recently, a novel coronavirus, SARS-CoV-2, caused a still ongoing pandemic. Epidemiological study suggested this virus was associated with a wet market in Wuhan, China. However, the exact source of this virus is still unknown. In this study, we attempted to assemble the complete genome of a coronavirus identified from two groups of sick Malayan pangolins, which were likely to be smuggled for black market trade. The molecular and evolutionary analyses showed that this pangolin coronavirus we assembled was genetically associated with the SARS-CoV-2 but was not likely its precursor. This study suggested that pangolins are natural hosts of coronaviruses. Determining the spectrum of coronaviruses in pangolins can help understand the natural history of coronaviruses in wildlife and at the animal-human interface, and facilitate the prevention and control of coronavirus-associated emerging diseases.

## Introduction

In December 2019, there was an outbreak of pneumonia with an unknown cause in Wuhan, Hubei province, China, with an epidemiological link to the Huanan Seafood Wholesale Market, a local live animal and seafood market. Clinical presentations of this disease greatly resembled viral pneumonia. Through deep sequencing on the lower respiratory tract samples of patients, a novel coronavirus named the 2019 novel coronavirus was identified [1], the name of which was then determined as SARS-CoV-2. This virus has spread to all provinces across China and more than 200 additional countries. As of April 11, 2020, the epidemic has resulted in 83,400 laboratory confirmed cases, 3,349 of which were fatal in China, while there were 1,643,047 laboratory confirmed cases and 101,507 deaths in other countries. The global toll of new cases and deaths is still increasing sharply.

To effectively control the disease and prevent new spillovers, it is critical to identify the animal origin of this newly emerging coronavirus. In the Wuhan wet market, high viral loads were reported in environmental samples. However, a variety of animals, including wildlife, were sold in this market, and the daily number and species of animals were very dynamic. Therefore, it remains unclear which animals initiated the first infections.

Coronaviruses usually cause respiratory and gastrointestinal tract infections and are genetically classified into four major genera: *Alphacoronavirus*, *Betacoronavirus*, *Gammacoronavirus*, and *Deltacoronavirus*. The former two genera primarily infect mammals, whereas the latter two predominantly infect birds [2]. In addition to SARS-CoV-2, other members of the *Betacoronavirus* genus caused the 2003 SARS (severe acute respiratory syndrome) outbreaks and the 2012 MERS (Middle East respiratory syndrome) outbreaks in humans [3, 4]. SARS-CoV and MERS-CoV are of bat origin, but both coronaviruses had an intermediate host: palm civets for SARS-CoV [5] and dromedary camels for MERS-CoV [6].

Approximately 30,000 base pairs in the coronavirus genome code for up to 11 proteins, including the surface glycoprotein Spike (S) protein binds to receptors on the host cell, which initiates virus infection. Different coronaviruses can use distinct host receptors due to structural variations in the receptor binding domains of their virus S protein. SARS-CoV uses angiotensin-converting enzyme 2 (ACE2) as one of the main receptors [7] with CD209L as an alternative receptor [8], whereas MERS-CoV uses dipeptidyl peptidase 4 (DPP4, also known as CD26) as the primary receptor. A recent study demonstrated that SARS-CoV-2 uses the SARS-CoV receptor ACE2 for entry and the serine protease TMPRSS2 for S protein priming [9].

Soon after the release of the SARS-CoV-2 genome, a scientist released a full genome of a coronavirus, Bat-CoV-RaTG13, from the bat species *Rhinolophus affinis*, which was colonized in Yunan province, nearly 2,000 km away from Wuhan. Bat-CoV-RaTG13 was 96% identical at the whole genome level to the SARS-CoV-2, suggesting the SARS-CoV-2 could be of bat origin [1]. However, because direct human-bat contact is rare, it seems to be more likely that the spillover of SARS-CoV-2 to humans from an intermediate host rather than directly from bats, as was the cases with both SARS-CoV and MERS-CoV.

The goal of this study was to determine the genetic relationship between a coronavirus from two groups of sick pangolins and SARS-CoV-2, and to assess whether pangolins could be potential intermediate hosts of SARS-CoV-2.

## Results

In March and July of 2019, we detected *Betacoronaviruses* in three individuals from two sets of smuggled Malayan pangolins (*Manis javanica*) (n = 27) that were intercepted by Guangdong customs [10]. All three animals suffered from serious respiratory disease and failed to be rescued by the Guangdong Wildlife Rescue Center [10] (S1 Table). Through metagenomic sequencing and *de novo* assembling, we recovered 38 contigs ranging from 380 to 3,377 nucleotides, and the nucleotide sequence identity among the contigs from these three samples were 99.54%. Thus, we pooled sequences from three samples and assembled the draft genome of this pangolin origin coronavirus. After that, gap filling with amplicon sequencing was conducted to obtain a nearly full genome sequence. This pangolin-CoV-2020 genome (Genbank No.: MT121216) was found to be comprised of 29,521 nucleotides.

**Fig 1:**
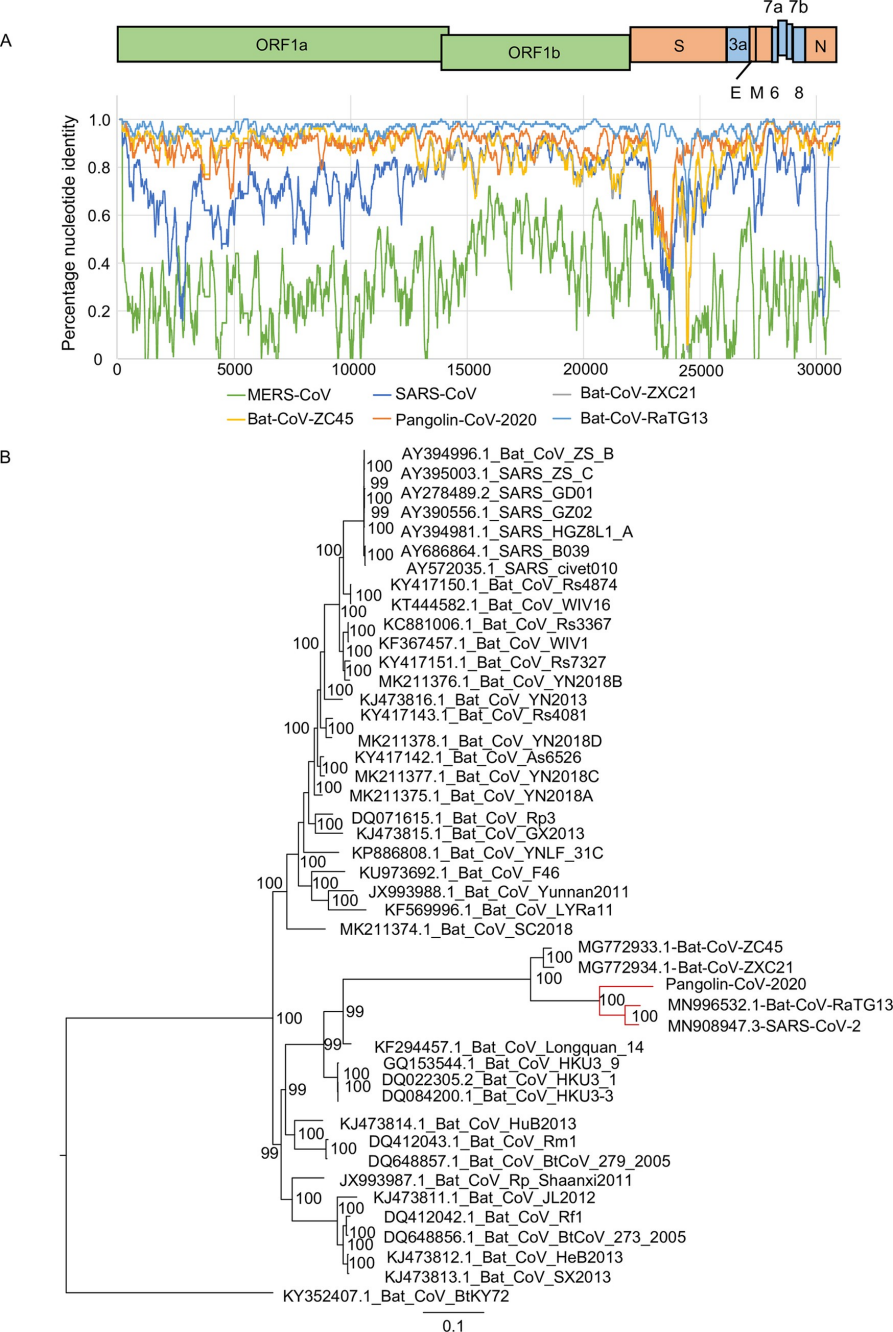
Genomic comparison of pangolin-CoV-2020, SARS-CoV-2, and other coronaviruses. A) Similarity plot based on the full-length genome sequence of SARS-CoV-2. Full-length genome sequences of Bat-CoV-RaTG13, Bat-CoV-ZXC21, SARS-CoV, Bat-CoV-ZC45, MERS-CoV, and pangolin-CoV-2020 were used as subject sequences. The green line indicates MERS-CoV, the dark blue line indicates SARS-CoV, the grey line indicates Bat-CoV-ZXC21, the yellow line indicates Bat-CoV-ZC45, the orange line indicates pangolin-CoV-2020, while the light blue line indicates Bat-CoV-RaTG13; B) Phylogenetic analyses of whole genome sequences depicting the evolutionary relationship among SARS-CoV-2, pangolin-CoV-2020, and other coronaviruses from different hosts. The phylogenies were estimated using the MrBayes approach employing the GTR+I+G nucleotide substitution model.

Strikingly, genomic analyses suggested the pangolin-CoV-2020 has a high identity with both SARS-CoV-2 and Bat-CoV-RaTG13, the proposed origin of SARS-CoV-2 (Fig 1A, S2 Table). The nucleotide sequence identity between pangolin-CoV-2020 and SARS-CoV-2 was 90.32%, whereas the protein sequence identity for individual proteins can be up to 100% (Table 1; Table 2). The nucleotide sequence identity between pangolin-CoV-2020 and Bat-CoV-RaTG13 was 90.24%, while that for the corresponding regions between SARS-CoV-2 and Bat-CoV-RaTG13 was 96.18% (Table 1, S1 Table).

**Table 1:** Nucleotide sequence identity among the whole genome, each gene or region of pangolin-CoV-2020 and other representative coronavirus against SARS-CoV-2.

	Nucleotide sequence identity (%)													
	Whole genome	S	RBD	E	M	N	ORF1ab	RdRp	ORF3a	ORF6	ORF7a	ORF7b	ORF8	ORF10
Pangolin-CoV-2020	90.32	84.52	86.64	99.11	93.24	96.18	90.36	91.31	93.21	95.70	93.39	91.47	91.82	99.15
Bat-CoV-RaTG13	96.18	93.15	86.19	99.56	95.93	96.90	96.52	97.80	96.24	98.39	95.59	99.22	96.99	99.15
Bat-CoV-ZXC21	88.04	76.74	67.32	86.67	93.39	91.17	89.12	86.99	88.85	95.16	89.62	95.35	88.53	100.00
Bat-CoV-ZC45	88.06	77.14	68.64	86.67	93.39	91.09	89.15	86.70	87.76	95.16	89.31	94.57	88.53	99.15
SARS-CoV	79.75	74.05	73.30	94.67	84.92	88.62	80.02	88.58	75.67	76.88	82.65	86.18	52.87	93.16

**Table 2:** Protein sequence identity among the whole genome, each gene or region of pangolin-CoV-2020 and other representative coronaviruses against SARS-CoV-2.

	Amino acid sequence identity (%)													
	Whole genome	S	RBD	E	M	N	ORF1ab	RdRp	ORF3a	ORF6	ORF7a	ORF7b	ORF8	ORF10
Pangolin-CoV-2020	96.00	90.18	96.80	100.00	98.18	97.83	96.73	99.35	97.05	96.67	97.49	95.24	94.12	97.33
Bat-CoV-RaTG13	98.43	97.69	89.56	100.00	99.09	99.04	98.55	99.57	97.79	100.00	97.49	97.65	94.91	97.33
Bat-CoV-ZXC21	93.45	79.66	66.35	100.00	98.64	94.10	95.56	95.69	91.66	93.22	87.70	92.77	94.04	100.00
Bat-CoV-ZC45	93.59	80.36	66.35	100.00	98.64	94.10	95.71	96.03	90.47	93.22	86.77	92.77	94.04	97.33
SARS-CoV	83.39	74.54	70.17	95.92	89.01	90.49	85.57	96.48	68.02	62.68	84.86	84.18	40.00	82.31

**Fig 2:**
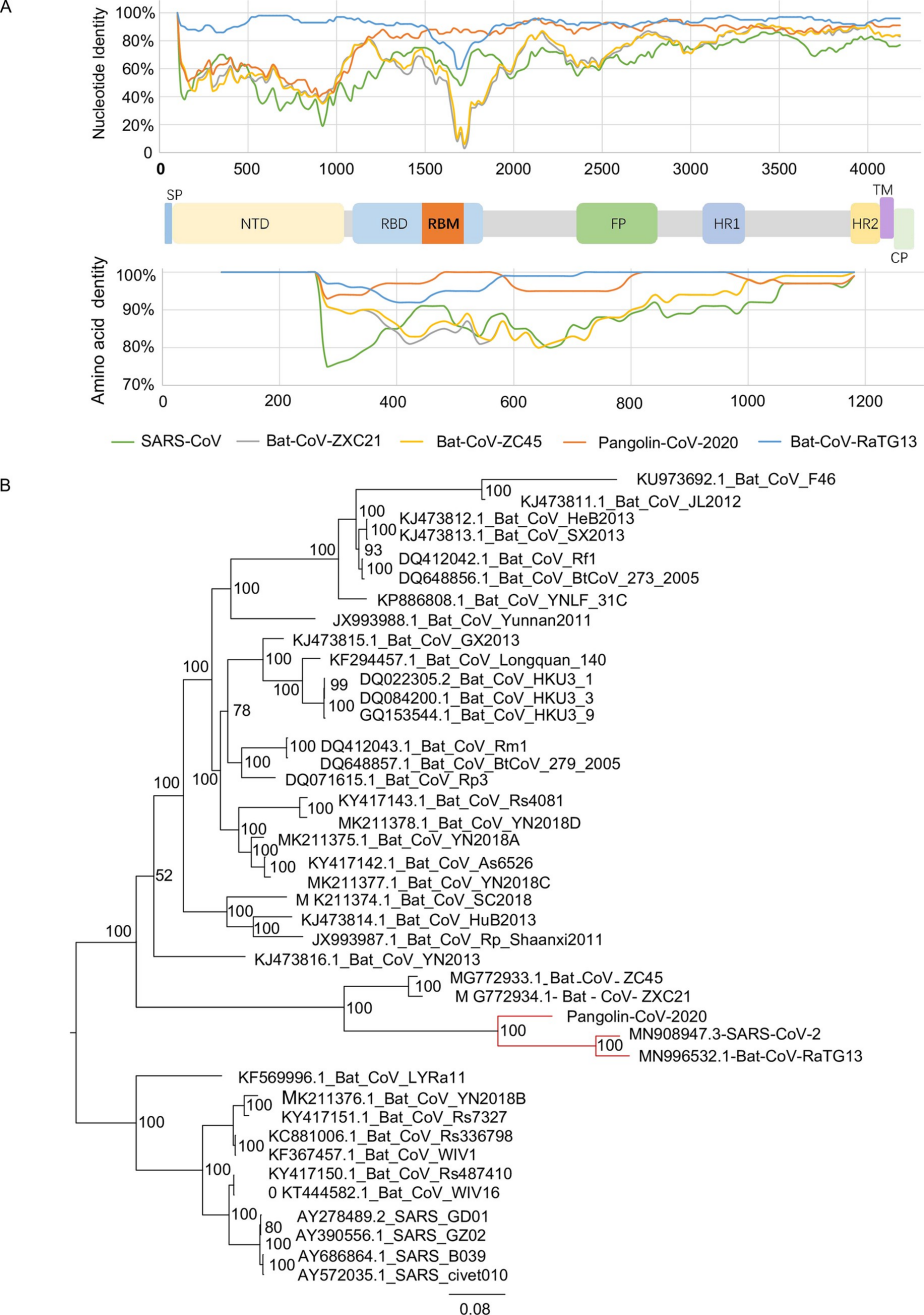
Genetic analyses of the spike (S) surface glycoprotein of pangolin-CoV-2020, SARS-CoV-2, and other coronaviruses. A) Similarity plot based on the spike surface glycoprotein amino acid and nucleotide sequence of SARS-CoV-2. Bat-CoV-RaTG13, Bat-CoV-ZXC21, Bat-CoV-ZC45, SARS-CoV, and pangolin-CoV-2020 were used as subject sequences. The green lines indicate SARS-CoV, the grey lines indicate Bat-CoV-ZXC21, the yellow lines indicate Bat-CoV-ZC45, the orange lines indicate pangolin-CoV-2020, while the light blue lines indicate Bat-CoV-RaTG13; B) Phylogenetic analysis of S gene sequences depicting the evolutionary relationship among SARS-CoV-2, pangolin-CoV-2020, and other coronaviruses from different hosts. The phylogenies were estimated using MrBayes approach employing the GTR+I+G nucleotide substitution model.

The nucleotide sequence identities among the S protein genes were 93.15% between the Bat-CoV-RaTG13 and SARS-CoV-2, 84.52% between pangolin-CoV-2020 and SARS-CoV-2, as well as 73.43% between pangolin-CoV-2020 and SARS-CoV, respectively (Table 1). Further analyses suggested the S gene was relatively more genetically diverse in the S1 region than the S2 region (Fig 2A, S3 Table). Compared with their nucleotide sequences, the S proteins of pangolin-CoV-2020 and SARS-CoV-2 were more conserved, with a sequence identity of 90.18% (Table 2).

**Fig 3:**
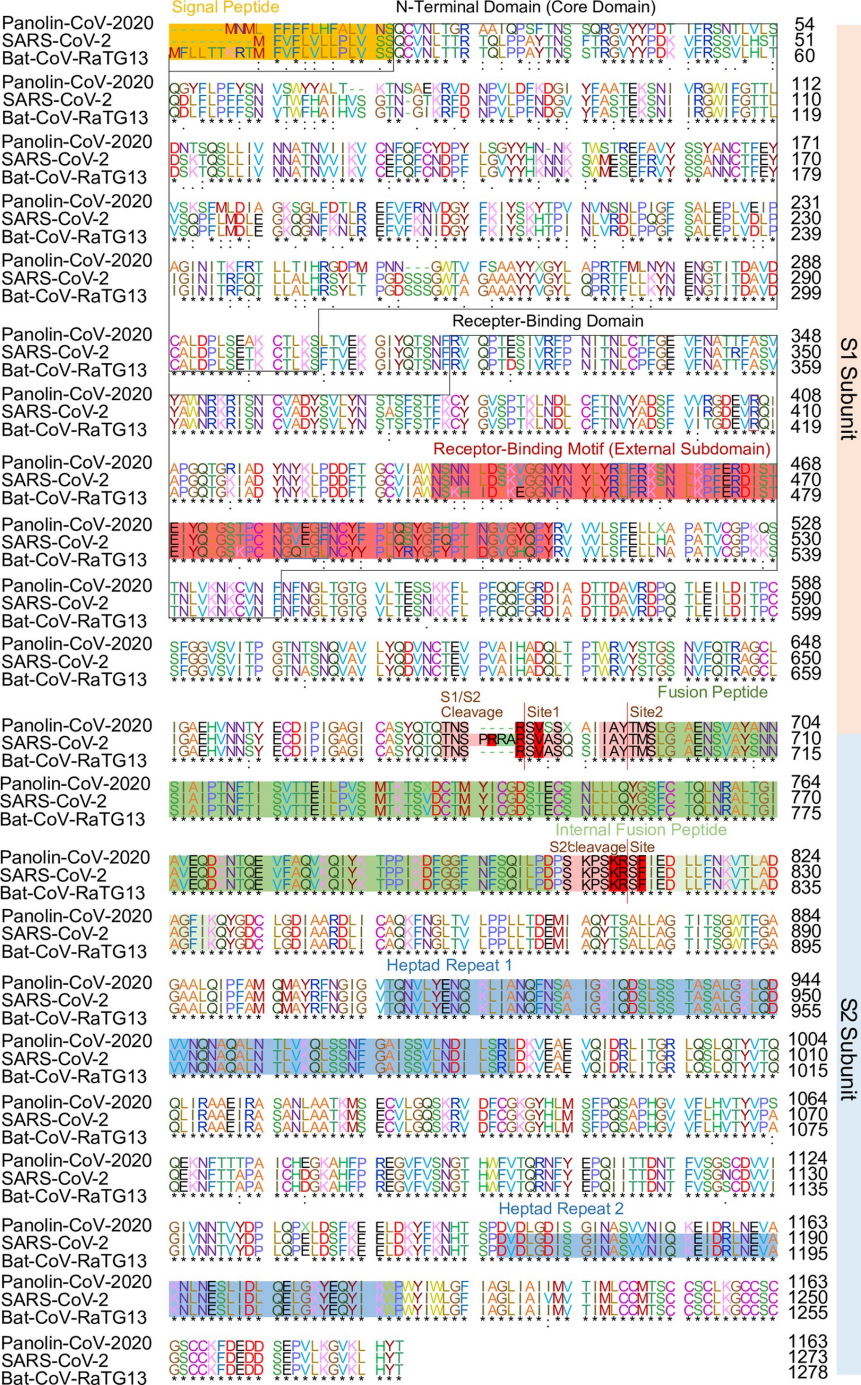
Amino acid sequence alignment of the spike (S) surface glycoprotein of the pangolin-CoV-2020 with SARS-CoV-2 and Bat-CoV-RaTG13. Previously identified critical ACE2-binding residues are in the blue box. An arginine in the core structure that interacts with glycan is displayed within the red box.

The receptor binding domains (RBD) of the S protein were highly conserved between pangolin-CoV-2020 and SARS-CoV-2, the nucleotide and amino acid sequences identity of RBD of S gene between them was highest in comparison with those between pangolin-CoV-2020 and other SARS-like conronaviruses of 86.64% and 96.80% (Table 1, Table 2). Pangolin-CoV-2020 and SARS-CoV-2 also shared a very conserved receptor binding motif (RBM) (98.6%), which was more conserved than in Bat-CoV-RaTG13 (76.4%) (Fig 3). These results support that pangolin-CoV-2020 and SARS-CoV-2 share the same angiotensin-converting enzyme 2 (ACE2) receptor. Further analyses suggested that there was one variation (Gln498) between the RBM of pangolin-CoV-2020 and that of SARS-CoV-2 but conserved in all other key residues being associated with receptor binding (Gly482, Val483, Glu484, Gly485, Phe486, Gln493, Leu455, Asn501), suggesting a potential binding affinity between pangolin-CoV-2020 and human ACE2 receptor (Fig 3).

On the other hand, unlike RBD, the nucleotide and amino acid sequence identity of NTD (N-terminal domain) were only 66.2% and 63.1% identical between pangolin-CoV-2020 and SARS-CoV-2. However, a loci Arg408 from the RBD core of SARS-CoV-2 could form a hydrogen bond with human ACE2 was conserved in pangolin-CoV-2020 (Fig 3). Both pangolin-CoV-2020 and Bat-CoV-RaTG13 lack an S1/S2 cleavage site (~680–690 aa) whereas SARS-CoV-2 possesses (Fig 3).

Genomic analyses suggested sequence similarities were not homogeneous across the S genes of pangolin-CoV-2020, SARS-CoV-2, Bat-CoV-ZXC21 and Bat-CoV-ZC45. For example, the first S region (i.e., nucleotides 1–1200) of pangolin-CoV-2020 has a higher nucleotide identity to two bat viruses (Bat-CoV-ZXC21 and Bat-CoV-ZC45) than SARS-CoV-2 and Bat-CoV-RaTG13, whereas the remaining S gene of pangolin-CoV-2020 is opposite (Fig 2A). These results suggest that a recombination event could have occurred during the evolution of these coronaviruses.

Phylogenetic analyses suggested that the S genes of pangolin-CoV-2020, SARS-CoV-2 and three bat origin coronaviruses (Bat-CoV-RaTG13, Bat-CoV-ZXC21, and Bat-CoV-ZC45) were genetically more similar to each other than other viruses in the same family (Fig 2B). The S gene of Bat-CoV-RaTG13 was genetically closer to pangolin-CoV-2020 than Bat-CoV-ZXC21 and Bat-CoV-ZC45. Similar tree topologies were observed for the encoding ORFs of RNA-dependent RNA polymerase (RdRp gene) and other genes (S1–S3 Figs). At the genomic level, SARS-CoV-2 was also genetically closer to Bat-CoV-RaTG13 than pangolin-CoV-2020 (Fig 1B).

## Discussion

In this study, we assembled the genomes of coronaviruses identified in sick pangolins and our results showed that pangolin-CoV-2020 is genetically associated with both SARS-CoV-2 and a group of bat coronaviruses. There is a high sequence identity between pangolin-CoV-2020 and SARS-CoV-2. However, phylogenetic analyses and a special amino acid sequence in the S gene of SARS-CoV-2 did not support the hypothesis of SARS-CoV-2 arising directly from the pangolin-CoV-2020.

It is of interest that the genomic sequences of coronaviruses detected from two batches of smuggled pangolins intercepted by different customs at different dates were all be associated with bat coronaviruses. In addition, the genetic identity of coronavirus contigs assembled in each animal was extremely high (99.54%). The reads from the third pangolin acquired in July 2019 were relatively less abundant than those from the two pangolin samples acquired in March 2019. Although it is unclear whether coronaviruses in these two batches of smuggled pangolins had the same origin, our results indicated that the pangolins can be a natural host for *Betacoronaviruses*, which could be enzootic in pangolins.

All three exotic pangolins detected with *Betacoronavirus*es were sick with serious respiratory diseases and failed to be rescued. However, these pangolins were very stressful in the transportation freight when being intercepted by the customs. It is unclear whether this coronavirus is a common virus flora in the respiratory tract of pangolins. Nevertheless, the pathogenesis of this coronavirus in pangolins remains to be elucidated.

Phylogenetic trees suggested that Bat-CoV-RaTG13 was more genetically close to SARS-CoV-2 at both individual gene and genomic sequence level compared with the genomic sequence of pangolin-CoV-2020 assembled in this study. Recombination analysis showed that S gene of pangolin-CoV-2020 might be constructed by fragment from Bat-CoV-ZC45 or Bat-CoV-ZXC21 and fragment from Bat-CoV-RaTG13. Interestingly, the cleavage site between S1 and S2 in SARS-CoV-2 had multiple insertions (i.e. PRRA), compared with those of Bat-CoV-RaTG13 and pangolin-CoV-2020, which may result from an additional recombination event. A new study reported a novel bat-derived coronavirus (RmYN02) identified from a metagenomics analysis of samples from 227 bats collected from the Yunnan province in China between May and October of 2019. Although RmYN02 showed a relatively low nucleotide sequence identity (93.3%) to SARS-CoV-2, it had a similar manner of the insertion of multiple amino acids at the junction site of the S1 and S2 subunits of the S protein as SARS-CoV-2, providing strong evidence that such insertion events can occur in nature [11]. Thus, these data suggest that SARS-CoV-2 originated from multiple naturally occurring recombination events among viruses present in bats and other wildlife species.

The S protein of coronaviruses binds to host receptors via RBDs and plays an essential role in initiating viral infection and determining host tropism [2]. A prior study suggested that SARS-CoV-2 and SARS-CoV bind to the same ACE2 receptor [9]. Our analyses showed that pangolin-CoV-2020 has a much conserved RBD to these viruses compared to MERS-CoV, suggesting that pangolin-CoV is very likely to use ACE2 as its receptor as well. A comparative analysis of the interaction of the S proteins of coronaviruses with ACE2 proteins of humans and pangolins showed that the S proteins of SARS-CoV-2 and pangolin-CoV can potentially recognize ACE2 in both humans and pangolins [12]. A recent study found that a human ACE2-binding ridge in SARS-CoV-2 RBD takes a more compact conformation compared with the SARS-CoV RBD; moreover, several residue changes in SARS-CoV-2 RBD may also enhance its human ACE2-binding affinity [13]. The core residues in RBM which may related to higher human ACE2-binding affinity than SARS-CoV are 100% identical between SARS-CoV-2 and CoV-Pangolin-2020. Therefore, pangolin-CoV-2020 (CoV-pangolin/GD) potentially recognizes human ACE2 better than the SARS-CoV.

In addition to RBD, NTD is also important in recognizing acetylated sialic acids on glycosylated cell-surface receptors [14]. It is reported that SARS-CoV-2 can bind to human ACE2 via the viral CTD (the same as RBD), but not NTD, and that the glycan attached to Asn90 from human ACE2 forms a hydrogen bond with Arg408 from the RBD core [15]. This glycan interacting Arginine is conserved between SARS-CoV-2 and pangolin-CoV-2. Therefore, there is structural similarity in glycan binding between SARS-CoV-2 and pangolin-CoV-2020. On the other hand, ACE2 receptor is present in pangolins with a high sequence conservation with those in the gene homolog in humans. However, the zoonosis of pangolin-CoV-2020 remains unclear.

The coronaviruses are shown to have a wide range of hosts, and some of them can infect humans [16]. Thus, it is critical to determine the natural reservoir and the host tropisms of these coronaviruses, especially their potential of causing zoonosis. In the last two decades, apart from SARS-CoV-2, SARS and MERS have caused serious outbreaks in humans, leading to thousands of deaths [3, 4, 17, 18]. Although these three zoonotic coronaviruses were shown to be of bat origin, they seemed to use different intermediate hosts. For example, farmed palm civets were suggested to be an intermediate host for SARS-CoV, although the details of the link from bats to farmed palm civets remain unclear [19–21]. Most recently, dromedary camels in Saudi Arabia were shown to harbor three different coronaviruses, including the dominant MERS-CoV lineage that was responsible for the outbreaks in the Middle East and South Korea during 2015 [22]. Although this present study does not support that pangolins would be intermediate hosts for the emergence of SARS-CoV-2, our results do not exclude the possibility that other CoVs could be circulating in pangolins. Thus, surveillance of coronaviruses in pangolins could improve our understanding of the spectrum of coronaviruses in pangolins. In addition to conservation of wildlife, minimizing the exposures of humans to wildlife will be important to reduce the spillover risks coronaviruses from wild animals to humans.

In summary, we suggest that pangolins could be natural hosts of *Betacoronaviruses* with an unknown potential to infect humans. However, our study does not support that SARS-CoV-2 evolved directly from the pangolin-CoV.

## Materials and methods

### Ethics statement

The study design was approved by the ethics committee for animal experiments at the Guangdong Institute of Applied Biological Resources (reference number: GIABR20170720; 20 July 2017) and followed basic principles outlined by this committee.

### Data selection

During our routine wildlife rescue efforts, one of the goals was to identify pathogens causing wildlife diseases. In 2019, we were involved in two events of pangolin rescues: one involved with 21 smuggling pangolins in March and the second with 6 smuggling pangolins in July. Although extensive rescue efforts were made, the majority of thse pangolins were dead. Most of the dead pangolins had a swollen lung, which contained a frothy liquid, and symptoms of pulmonary fibrosis. In the minority of these dead ones, we observed hepatomegaly and splenomegaly. From 11 pangolins failed to be rescued, we collected samples from their lung, lymph and spleen tissues and subjected for metagenomic analyses. Coronaviruses were detected in three individuals by mapping clean reads without ribosomes and host sequences to an in-house virus reference dataset separated from the GenBank non-redundant nucleotide database using the Burrows-Wheeler Aligner (BWA) ver 0.7.17 [10, 23]. Two of these animals were from the first batch of smuggled Malayan pangolins intercepted by Meizhou, Yangjiang, and Jiangmen customs in March, 2019, and the third one was from the second batch in a freight being transported from Qingyuan to Heyuan in July, 2019. The RNA samples from these three individuals were subjected to deep sequencing.

### Genomic assembly and sequence analyses

Clean reads from each of the three coronavirus positive animals were *de novo* assembled using MEGAHIT v1.2.9 [24]. After examining the high similarity of 99.54% among the samples from three animals, to maximize the coverage of the virus genome, we pooled clean reads and *de novo* assembled them. The assembled contigs were used as references for extracting unmapped reads using Salmon v0.14.1 [25], and multiple rounds were implemented to maximize the mapping.

A total of 38 contigs were identified to be highly similar to the SARS-CoV-2 genome using BLASTn and tBLASTx. GapFiller v1.10 and SSPACE v3.0 were used to fill gaps and draft pangolin-CoV-2020 genome was constructed with ABACAS v1.3.1 (http://abacas.sourceforge.net/) [26–28].

Gaps in the draft genomes were filled using the 2x PCR Mix (Gentech, China) by reverse transcription PCR (RT-PCR). Primers were designed based on the draft genome sequence of the pangolin-CoV-2020 we assembled (S4 Table). After gap filling, the whole genome sequence of pangolin-CoV-2020 was submitted to GenBank databases (accession no. MT121216).

Multiple sequence alignments were conducted using MUSCLE [29]. Changing patterns of sequence identity were analyzed using SimPlot v3.5.1 to determine the sequence identity among SARS-CoV-2 (MN908947.3), pangolin-CoV-2020, Bat-CoV-RaTG13 (MN996532.1), Bat-CoV-ZXC21 (MG772934.1), Bat-CoV-ZC45 (MG772933.1), SARS-CoV (AY395003.1), and MERS-CoV (NC_019843.3) at both the genomic sequence level and the individual gene level [30]. The sequence identity between the whole genome and different genes or regions was calculated utilizing p-distance in MEGA v10.1.7 [31].

### Phylogenetic analyses and recombination

We downloaded 44 full-length genome sequences of coronaviruses isolated from different hosts from the public database (S5 Table), with the data kindly shared by the submitters. Phylogenetic analyses were performed based on their whole genome sequences, RdRp gene, S gene, small envelope protein (E gene), as well as all other gene sequences. We constructed multiple sequence alignments of their complete genomes and individual genes using MAFFT v7.407 [32]. Phylogenetic analyses were estimated using MrBayes [33] with 50,000,000 generations and the 25% of the generations as burnin. The best models were determined by jModelTest v2.1.7 [34]. Then, the trees were visualized and exported as vector diagrams with FigTree v1.4.4 (http://tree.bio.ed.ac.uk/software/figtree/). Potential recombination events and the location of possible breakpoints in coronavirus genomes were detected using SimPlot v3.5.1 [30].

## Supporting information

S1 Table Number of sequencing reads assigned to different viruses in each pangolin sample. We only focused on individual samples with coronavirus reads in this study.
(XLSX)

S2 Table Nucleotide sequence identity of SARS-CoV-2, pangolin-CoV-2020, Bat-CoV-RaTG13, Bat-CoV-ZXC21, Bat-CoV-ZC45, SARS-CoV, and MERS-CoV in 200 bp windows with an overlap of 20 bp. SARS-CoV-2 was considered as query, while the other five viruses were used as references.
(XLSX)

S3 Table Nucleotide and amino acid sequence identity of SARS-CoV-2, pangolin-CoV-2020, Bat-CoV-RaTG13, Bat-CoV-ZXC21, Bat-CoV-ZC45, and SARS-CoV in 200 bp windows with an overlap of 20 bp. SARS-CoV-2 was considered as query, while the other four viruses were used as references.
(XLSX)

S4 Table Information on RT-PCR primers filling gaps in genome sequences. (XLSX)

S5 Table Accession numbers and strain IDs of coronavirus strains isolated from different hosts. (XLSX)

S1 Fig Phylogenetic analyses of gene sequences depicting the evolutionary relationship between SARS-CoV-2, pangolin-CoV-2020, and other coronaviruses from different hosts using the MrBayes approach: A) small envelope gene sequences employing the HKY+G nucleotide substitution model, B) RNA-dependent RNA polymerase (RdRp) sequences employing the GTR+I+G nucleotide substitution model, C) matrix protein sequences employing the GTR+I+G nucleotide substitution model, D) nucleocapsid protein sequences employing the GTR+I+G nucleotide substitution model.
(TIF)

S2 Fig Phylogenetic analyses of gene sequences depicting the evolutionary relationship between SARS-CoV-2, pangolin-CoV-2020, and other coronaviruses from different hosts using the MrBayes approach: A) ORF1ab gene sequences employing the GTR+I+G nucleotide substitution model, B) ORF3a gene sequences employing the GTR+I+G nucleotide substitution model, C) ORF6 gene sequences employing the HKY+G nucleotide substitution model.
(TIF)

S3 Fig Phylogenetic analyses of gene sequences depicting the evolutionary relationship between SARS-CoV-2, pangolin-CoV-2020, and other coronaviruses from different hosts using the MrBayes approach: A)ORF7a gene sequences employing the GTR+G nucleotide substitution model, B) ORF7b gene sequences employing the HKY+G nucleotide substitution model, C) ORF8 gene sequences employing the GTR+G nucleotide substitution model, and D) ORF10 gene sequences employing the HKY+G nucleotide substitution model.
(TIF)
