# Correction: Are pangolins the intermediate host of the 2019 novel coronavirus (SARS-CoV-2)?

**DOI:** 10.1371/journal.ppat.1009664

**Published:** 2021-06-09

**Authors:** Ping Liu, Jing-Zhe Jiang, Xiu-Feng Wan, Yan Hua, Linmiao Li, Jiabin Zhou, Xiaohu Wang, Fanghui Hou, Jing Chen, Jiejian Zou, Jinping Chen

After this article [1] was published, concerns were raised about the availability of the underlying data and the relationship of this work to similar studies reported in *Viruses* and *Nature* [2, 3]. The concerns and the study reported in [1] were assessed by the journal with input from three members of *PLOS Pathogens*’ Editorial Board and an external reviewer. The consulted editors and reviewer advised that the article’s conclusions are valid and are supported by the results, although aspects of the study were not adequately reported in the original publication. These issues, and an update to the Data Availability statement, are addressed in this notice.

Two samples included in the *PLOS Pathogens* study overlapped with samples and data used for the study reported in [2]. The sample IDs of these two samples were lung07, lung08, and the raw data for these samples are available through NCBI accession number PRJNA573298, BioSample IDs SAMN12809952 and SAMN12809953. The third sample reported in [1] was newly reported in the *PLOS Pathogens* article, the raw data for this sample are available under NCBI accession number PRJNA686836, BioSample ID SAMN17126166. All three samples used in [1] were obtained from the Guangdong Wildlife Rescue Center.

The raw data for the lung07 and lung08 samples (originally reported in [2]) appear also to have been used, but reported under different sample IDs, in [3]. (This has been discussed in [4].) Two researchers (JZ, FH) were listed authors on both [1] and [3]. For the *PLOS Pathogens* study these individuals were involved in obtaining samples from the Guangdong Wildlife Rescue Center; the Guangdong Wildlife Rescue Center also cooperated with the authors of the *Nature* article [3]. According to the corresponding author of [1], the other authors of the *PLOS Pathogens* article were unaware of the study reported in [3] until after the study reported in [1] had been completed.

Of note, both the *Nature* [3] and *PLOS Pathogens* [1] articles reported 90.3% whole genome sequence identity between pangolin coronavirus and SARS-CoV-2. This is lower than whole genome sequence identity between bat coronavirus (bat-CoV-RaTG13) and SARS-CoV-2 (96%). Though the receptor binding domain (RBD) of S gene was found to be more similar between pangolin coronavirus and SARS-CoV-2 than bat-CoV-RaTG13 and SARS-CoV-2, the similarities were not strong enough to support that pangolins are intermediate hosts of SARS-CoV-2.

FASTA sequences and the raw chromatograms for the PLOS Pathogens study’s gap-filling amplicons are provided here in S1 File. Pangolin specimens lung07 and lung08 were used for the gap closing by RT-PCR/Sanger sequencing. The lab records do not include details as to which of these results were obtained using lung07 versus lung08 samples as input, but most gaps were filled by lung08 sequence amplification. PCR products were sequenced from both ends, and each gap was filled with at least 2x coverage. The coronavirus-positive samples remain available for further validation studies. Interested researchers can contact the corresponding author for access.

The 2019 *Viruses* paper [2] relied only on short sequence reads; for that study, the authors were unable to report the complete genome because they did not have a SARS-CoV-2 reference sequence. After the SARS-CoV-2 complete sequence became available (GenBank accession number MN908947.3, [5–6]), the data included in [1] were reanalyzed using MN908947.3 as reference to obtain the complete pangolin-CoV-2020 assembly reported in the *PLOS Pathogens* article [1]. 38 contigs in [1] were different from 16 contigs published in [2]. The latter contigs [2] were assembled based on individual sample analyses, whereas the 38 contigs reported in [1] were obtained based on pooled sequences of three coronavirus positive samples (S2 File). After removing repeated contigs, there were 34 unique contigs ranging from 308bp (note: this was erroneously reported as 380bp in [1]) to 3377bp. One contig did not align to the newly assembled pangolin-CoV-2020 genome sequence, although it showed similarity to SARS-CoV-2 (MN908947.3) according to Blast. Therefore, 33 contigs were used in [1] to obtain the pangolin-CoV-2020 draft genome. The length of overlapping sequences ranged from 20 to 573bp, with most >200bp (S3 File). The contig sequences were aligned pairwise and sequences were removed if they aligned to unreasonable positions (e.g. an E gene sequence aligning to an S gene). This resulted in an average sequence identity of 99.54%, based on pairwise comparisons of all contigs, supporting the conclusion that these contigs were derived from the same virus strain.

An external reviewer for *PLOS Pathogens* evaluated the primary data that are included with this notice and deposited with NCBI. The reviewer confirmed that the MP789 genome reported in [1], and the article’s overall results, are supported by the primary data.

The viral read abundances in Supplemental Table 1 of [1] were calculated by Salmon software [7].

The *PLOS Pathogens* study [1] does have limitations. For example, it relies on sequencing data that were obtained from a small number of dead pangolin individuals. Further studies are needed to clarify whether the findings are representative of virus sequences present in wild pangolin populations.

The sequencing data accession numbers provided in the Data Availability Statement and Materials and Methods section of [1] are incorrect. A corrected Data Availability Statement is provided here:

The MP789 draft genome sequence of pangolin CoV is available from GenBank under accession number MT084071.1. GenBank accession number MT121216.1 is the whole genome without gaps. Raw data for the samples used in this study are deposited with NCBI under Project Numbers PRJNA573298 (BioSample IDs SAMN12809952 and SAMN12809953) and PRJNA686836 (BioSample ID SAMN17126166). Gap filling and contig sequences are provided in Supporting Information files with this notice.

## Supporting information

S1 File Sequences and chromatograms of gap filling. (RAR)

S2 File Sequences of contigs for assemble pangolin coronavirus. Among the 38 contigs, the sequences of contigs of k141_1468 flag = 1 multi = 3.0000 len = 525, k141_2756 flag = 1 multi = 3.0000 len = 525, and k141_7634 flag = 1 multi = 3.0000 len = 525; k141_5040 flag = 1 multi = 3.0000 len = 546 and k141_3865 flag = 1 multi = 3.0000 len = 546; as well as k141_15718 flag = 1 multi = 3.0000 len = 546 and k141_3865 flag = 1 multi = 3.0000 len = 546, had the same sequences. Contig k141_8467 flag = 1 multi = 6.0000 len = 345 did not align to the pangolin SARS-CoV-2 genome obtained in this study [1], and so it was not used in the reported genome assembly. Therefore, there were 33 unique contigs used to obtain pangolin CoV genome in total.
(TXT)

S3 File 33 final-contigs for assemble pangolin coronavirus, and the overlapped sequence length. We blasted each of the sequences in NCBI. For each, this file includes the Contig ID, Sequence, and the Subject ID, Start in contig sequences, End in contig sequences, Start in pangolin CoV genome, End in pangolin CoV genome, as well as Length of overlapped sequence (bp) for the top Blast hit.
(XLSX)
