# Supplementary figures and images for: Are pangolins the intermediate host of the 2019 novel coronavirus (SARS-CoV-2)?
Source: PLoS Pathog. 2020 May 14;16(5):e1008421. doi: 10.1371/journal.ppat.1008421 (PMC7224457; doi:10.1371/journal.ppat.1008421)

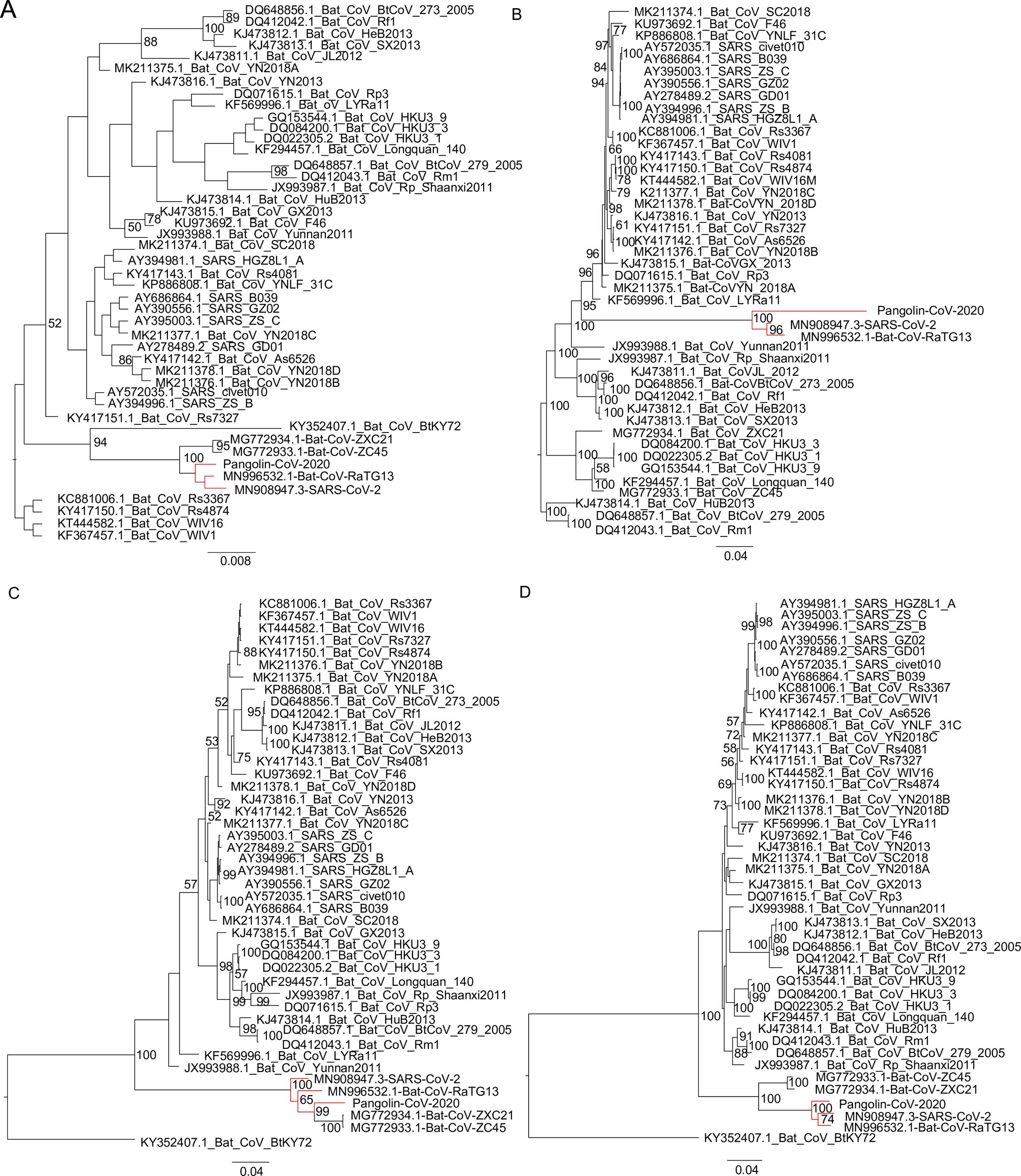

Supplement: S1 Fig — Phylogenetic analyses of gene sequences depicting the evolutionary relationship between SARS-CoV-2, pangolin-CoV-2020, and other coronaviruses from different hosts using the MrBayes approach: A) small envelope gene sequences employing the HKY+G nucleotide substitution model, B) RNA-dependent RNA polymerase (RdRp) sequences employing the GTR+I+G nucleotide substitution model, C) matrix protein sequences employing the GTR+I+G nucleotide substitution model, D) nucleocapsid protein sequences employing the GTR+I+G nucleotide substitution model. (TIF) [file ppat.1008421.s006.tif]

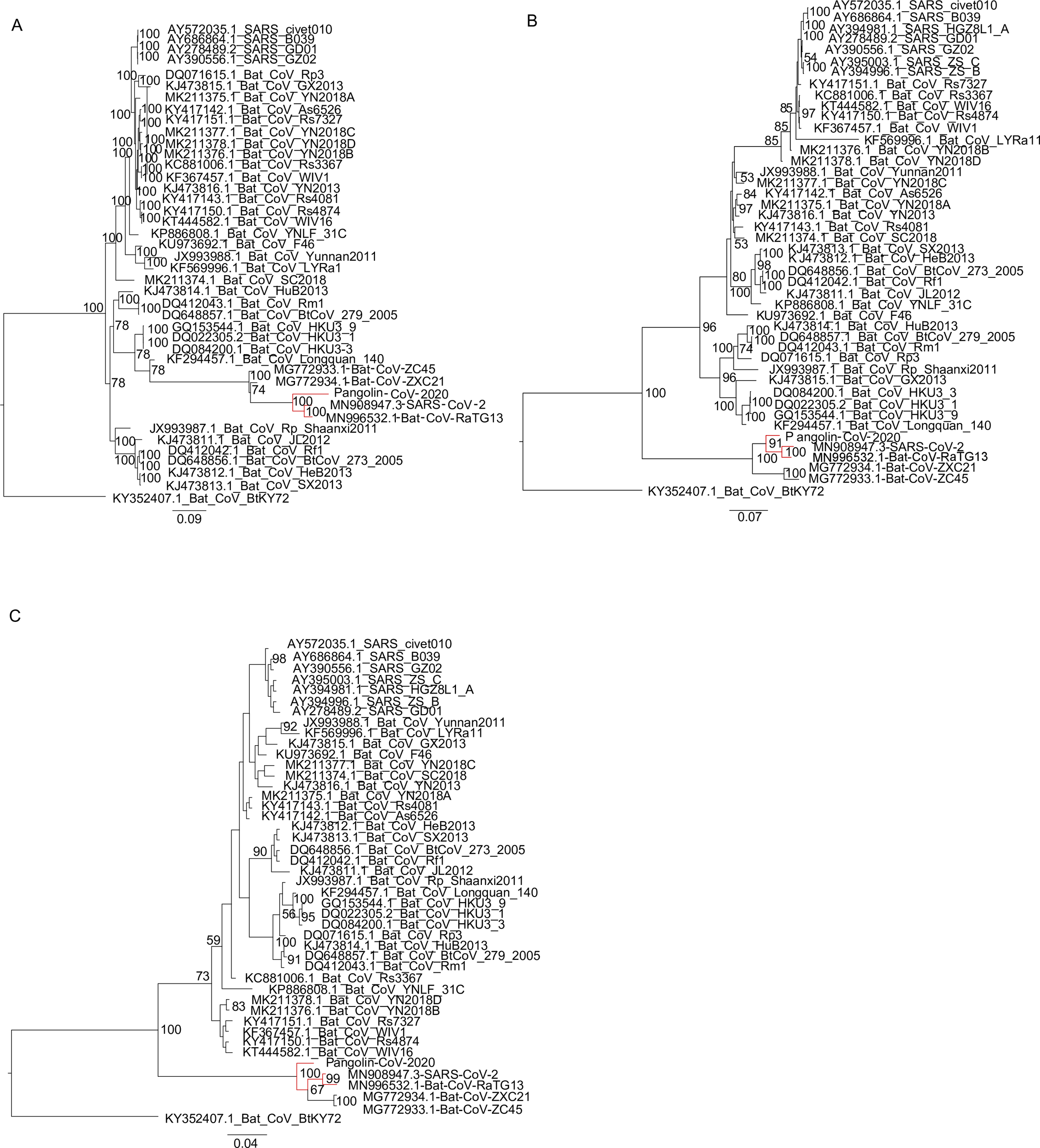

Supplement: S2 Fig — Phylogenetic analyses of gene sequences depicting the evolutionary relationship between SARS-CoV-2, pangolin-CoV-2020, and other coronaviruses from different hosts using the MrBayes approach: A) ORF1ab gene sequences employing the GTR+I+G nucleotide substitution model, B) ORF3a gene sequences employing the GTR+I+G nucleotide substitution model, C) ORF6 gene sequences employing the HKY+G nucleotide substitution model. (TIF) [file ppat.1008421.s007.tif]

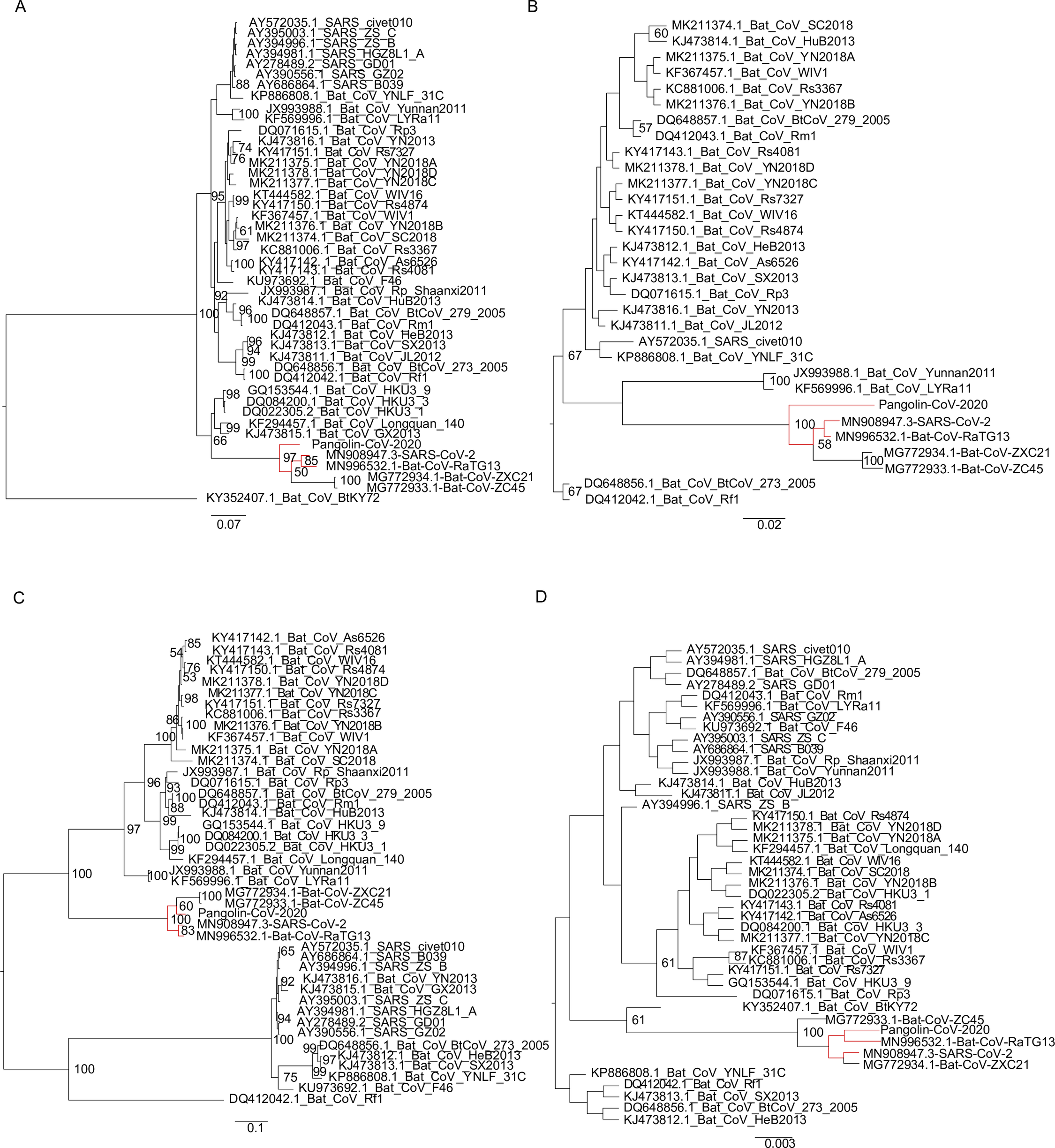

Supplement: S3 Fig — Phylogenetic analyses of gene sequences depicting the evolutionary relationship between SARS-CoV-2, pangolin-CoV-2020, and other coronaviruses from different hosts using the MrBayes approach: A)ORF7a gene sequences employing the GTR+G nucleotide substitution model, B) ORF7b gene sequences employing the HKY+G nucleotide substitution model, C) ORF8 gene sequences employing the GTR+G nucleotide substitution model, and D) ORF10 gene sequences employing the HKY+G nucleotide substitution model. (TIF) [file ppat.1008421.s008.tif]
